# Private Heat for Public Warmth: How Huddling Shapes Individual Thermogenic Responses of Rabbit Pups

**DOI:** 10.1371/journal.pone.0033553

**Published:** 2012-03-16

**Authors:** Caroline Gilbert, Dominic J. McCafferty, Sylvain Giroud, André Ancel, Stéphane Blanc

**Affiliations:** 1 Université Paris-Est, Ecole Nationale Vétérinaire d'Alfort, UMR 7179, CNRS, MNHN, Maisons-Alfort, France; 2 Institute of Biodiversity, Animal Health and Comparative Medicine, College of Medical, Veterinary & Life Sciences, University of Glasgow, Glasgow, United Kingdom; 3 Research Institute of Wildlife Ecology, University of Veterinary Medicine, Vienna, Austria; 4 Université de Strasbourg, IPHC, Strasbourg, France; 5 CNRS, UMR 7178, Strasbourg, France; Roehampton University, United Kingdom

## Abstract

**Background:**

Within their litter, young altricial mammals compete for energy (constraining growth and survival) but cooperate for warmth. The aim of this study was to examine the mechanisms by which huddling in altricial infants influences individual heat production and loss, while providing public warmth. Although considered as a textbook example, it is surprising to note that physiological mechanisms underlying huddling are still not fully characterised.

**Methodology/Principal Findings:**

The brown adipose tissue (BAT) contribution to energy output was assessed as a function of the ability of rabbit (*Oryctolagus cuniculus*) pups to huddle (placed in groups of 6 and 2, or isolated) and of their thermoregulatory capacities (non-insulated before 5 days old and insulated at ca. 10 days old). BAT contribution of pups exposed to cold was examined by combining techniques of infrared thermography (surface temperature), indirect calorimetry (total energy expenditure, TEE) and telemetry (body temperature). Through local heating, the huddle provided each pup whatever their age with an ambient “public warmth” in the cold, which particularly benefited non-insulated pups. Huddling allowed pups facing a progressive cold challenge to buffer the decreasing ambient temperature by delaying the activation of their thermogenic response, especially when fur-insulated. In this way, huddling permitted pups to effectively shift from a non-insulated to a pseudo-insulated thermal state while continuously allocating energy to growth. The high correlation between TEE and the difference in surface temperatures between BAT and back areas of the body reveals that energy loss for non-shivering thermogenesis is the major factor constraining the amount of energy allocated to growth in non-insulated altricial pups.

**Conclusions/Significance:**

By providing public warmth with minimal individual costs at a stage of life when pups are the most vulnerable, huddling buffers cold challenges and ensures a constant allocation of energy to growth by reducing BAT activation.

## Introduction

In shared developmental environments, competition for limited resources is a major mechanism shaping phenotypic differences among siblings [1]. However, at the same time that offspring within a nest compete for food supply, they jointly create an environment that promotes their growth [2]. The advantage of such a shared environment is especially noticeable in young altricial mammals for which thermal requirements in their first days determine their growth trajectories and survival (e.g. [3]–[7]). Huddling, an active and close aggregation of animals (review in [8]), is a widespread cooperative group behaviour that allows individuals to reduce their thermoregulatory expenses, thus enhancing survival when energy becomes a limiting factor. By huddling together in the nest, altricial pups then reduce their thermoregulatory costs and maintain a stable body temperature promoting growth [5]–[7], [9]–[11]. However, within a huddle, energy is allocated by each individual for thermogenesis, while the benefits resulting of group heating are shared. As pointed out by Haig [12], “heat generated by huddling animals is a public good with a private cost”.

As a response to cold, heat is first produced in young mammals by non-shivering thermogenesis (NST), depending on the activation of brown adipose tissue (BAT) [13]–[16]. Because NST rapidly generates a great amount of heat over a short period, it is an essential response to cold that prevents hypothermia in small mammals or newborns [17]. NST activation has, however, a cost. As an aerobic process very demanding in oxygen its acute use in altricial newborn will reduce growth rates whereas its chronic use will ultimately impact survival. Among altricial mammals, rat and rabbit newborns particularly face this trade-off. They are able to generate heat via NST in BAT in their first day of life but are unable to keep it due to the lack of body insulation and must rely on huddling to share the cost of thermoregulation [4], [10], [11], [18]–[22]. BAT in newborn rabbits is situated between the scapulae and around the neck; its mass is maximum at birth, and decreases by half during the first days of life [18]. This suggest that rabbit pups may gain a thermal benefit from their littermates' presence during the first five days of life when they are non-insulated [5], [6] but not later on. Thermoregulatory costs of rabbit pups may therefore depend both on their huddling behaviour and their changing developmental thermoregulatory constraints [6].

Since altricial mammals within their litter compete for energy but cooperate for warmth, we explored in this study the proximal thermoregulatory constraints that ultimately impact on their growth and survival in their litter. We predicted that the efficiency of huddling on the thermoregulatory responses of altricial rabbit pups facing a cold challenge will rapidly decrease as a function of age and thermoregulatory capacities i.e. insulated *vs.* non-insulated. In particular, we investigate how the huddle influences: 1) the thermal environment of the pup and 2) thermogenic responses of individuals as well as 3) how individual pups contribute to group thermoregulatory processes in the litter. In this context, we tested the physiological responses of rabbit pups in the cold from 23 to 11°C. By combining independent techniques to measure energy expenditure (indirect calorimetry), body temperature (implanted sensors) and surface temperature (infrared thermography [23]–[25]), we were able to evaluate the energetic contribution of non-shivering thermogenesis through BAT activation. The overall aim of this study is to provide information on how physiological constraints control thermoregulation in early life and its consequences for their life history.

## Materials and Methods

### Animals and housing conditions

The experiments were carried out on rabbits (*Oryctolagus cuniculus*) of a crossed strain, “Hyplus” from Grimaud (New Zealand White×Californian rabbits; Grimaud La Corbière, Roussay, France; http://www.grimaud.fr/). Four does and two males were housed individually in cages (50×100 cm, 50 cm high). Parturition occurred spontaneously after 30–31 days of gestation. The sliding door of a litter box (30×50 cm, 40 cm high) filled with fresh straw and hooked to the doe's cage was opened 3 days before parturition. Room temperature varied from 18 to 23°C, and a 16∶8-h light-dark cycle was maintained. The animals were kept and treated during experiments in accordance with the European Guidelines for Animal Care with full approvals from the French Government, the Centre National de la Recherche Scientifique and the Direction of the Veterinary Services (no. 67-188).

At birth, pups were left a few hours with the doe in the nesting box to allow them to suckle once without disturbance. Then pups were separated from the doe, color-marked on their back with an animal marking stick (Raidex, Dettingen/Erms, Germany) for identification, and weighed (Sartorius model 1403, Germany, www.sartorius.com/, ±0.1 g). Pups were placed in plastic boxes with an open top (28×42 cm, 16 cm high). Except during the experiment, all pups were housed in groups from their original litter in a room with a controlled ambient temperature of 23–24°C and continuous lighting. Ambient temperature set point was chosen to limit any mortality of the pups, based on a previous study by our team [6]. Suckling was allowed once a day, around 9:30–10:00 AM, and lasted for 3–4 min on average. Pups were weighed before and after suckling (±0.1 g) to monitor their growth. Experiments were undertaken during the afternoon, a few hours after suckling (*ca.* 4 hours), in order to minimize thermogenic effects due to post-prandial digestion [26]–[27].

### Composition of groups and cold exposure

Experiment 1 (n = 24 pups taken from four litters of 7–8 pups) was designed to explore thermoregulatory responses of pups. Pups were placed in a room adjacent to the breeding room, and exposed to decreasing ambient temperatures of 23 (for 2 hours), 18, 15, and 11°C (for 1 hour). Room temperature after the cold exposure was again regulated at 23–24°C. Non-insulated pups aged 4 days old were placed in groups of six (G6, 4 groups of 6 pups), and the subsequent day (at 5 days old), the same pups were separated in groups of two (G2, i.e. 12 groups of 2 pups). The same procedure was repeated when pups were 15 days old (G6 insulated pups; n = 4 G6 groups) and 16 days old (G2 insulated pups; n = 12 G2 groups). After the experiments all pups were placed in the breeding room.

Independently, Experiment 2 was designed to investigate brown adipose tissue activation using thermal imaging (n = 12 pups taken from two litters of 7–8 pups). Non-insulated pups aged 3 and 4 days old and insulated pups aged 10 and 11 days old were used. They were exposed to ambient temperatures of 23°C (for 2 hours) and 14°C (for 1 hour). On day 1, a group of six pups (G6, 3 days old) and two isolated pups (G1, 10 days old) were tested. On day 2, a group of six pups (G6, aged 11 days old, including the two G1 of day 1) and two isolated pups (G1, 4 days old, randomly selected from the G6 of day 1) were tested.

### Total energy expenditure (TEE)

Oxygen (O_2_) consumption and carbon dioxide (CO_2_) production were measured using an open-circuit respirometry system (Sable Systems International, Las Vegas, NV, USA). The concentrations of O_2_ and CO_2_ in the outgoing air were measured in one experimental chamber for G6 (at 4 and 15 days old, 25×36×16 cm, V = 14.4 L), and in three chambers for the three G2 (at 5 and 16 days old, 17×24×15 cm, V = 6.1 L). The two different sizes of chambers were defined after theoretical calculations taking into account the air flow and the energy expenditure of pups, to ensure robust respirometry measurements. Measurements were performed continuously over the cold challenge (from 23 to 11°C). Calibration of O_2_ and CO_2_ analyzers were undertaken before and after each experiment.

For the “G6-procedure”, the cage was sampled for 120 s (1 sample per second) at a flow rate of 1 L.min^−1^ every 4 min, while for the “G2-procedure”, the three cages were successively sampled for 120 s (1 sample per second) at a flow rate of 1 L.min^−1^ every 8 min. For both experiments, final values of O_2_ and CO_2_ concentrations were the mean of values recorded during 60 and 90 s, respectively. The system was flushed with air for 120 s between each cycle. Energy expenditure was calculated using an energy equivalent of 16.47 J.ml^−1^ of O_2_ consumed and 4.62 J.ml^−1^ of CO_2_ produced, according to Weir's equations [28]. Metabolic rate was expressed as kJ.day^−1^.g^−1^. The mean body mass of rabbits was determined from a weight before measurements and a weight the day after the experiment, before subsequent suckling.

For Experiment 2, the G6 was placed in a chamber of 25×36×16 cm, while the two G1 rabbit pups were placed in cages of 17×24×15 cm. The concentrations of O_2_ and CO_2_ in the outgoing air were measured simultaneously in the three chambers. Measurements were performed continuously over the cold challenge (at 23°C for 2 hours and 14°C for 1 hour). Samplings and calculations were similar to Experiment 1, according to “G2-procedure”.

### Microclimate within the calorimetric chambers

In order to evaluate any local heating effect, in particular in the chambers for G6 pups compared with G2 pups, temperatures were also measured in each calorimetric chamber. The ambient temperature within chambers was continuously monitored by a thermoresistive device (Pt-RTD 100, Jumo, Jumo-Regulation, Metz, France) placed in the centre of the chamber (see above for box dimensions). The thermal probes were connected to a Smart A/D computer card (model no. 619), and data were recorded every 30 s using Sensoray Quicksense software (version 3.3; Smart A/D and Quicksense, Sensoray, Tigard, OR; USA; http://www.sensoray.com). Thermoresistive devices were calibrated in a thermostatic bath from 20 to 40°C, with 5°C increments before and after the experiments.

### Body temperature

In Experiment 1, 11 randomly selected pups were implanted with transmitters at 2.5 days of age (mean body mass of *ca.* 85 g). TA10TA-F20 transmitters (Data Sciences International, St Paul, MN; USA; 3.5 g, 1.75 cm^3^) were placed intraperitoneally, under gaseous anesthesia (isoflurane, Forene) and strictly aseptic surgical conditions. The surgery took place at least 5 h after suckling, and a heating pad was used to prevent hypothermia. Antibiotics (oxytetracycline, Terramycine LA) and anti-inflammatory molecules (ketoprofen, Ketofen) were injected at the end of surgery and the pup was returned to its littermates within 1–2 h. Transmitters recorded body temperature at 30-s intervals during the experiments. Because of radio frequency interference, only one pup per group could be monitored (at 4 and 15 days old: 4 pups placed in G6; at 5 and 16 days old: 11 pups placed in G2). In Experiment 2, 4 randomly selected pups were implanted with transmitters following a similar protocol.

### Thermal images

In Experiment 2, the surface temperature of rabbit pups was recorded at the start and end of respirometry periods (at 23 and 14°C) using a thermal imaging camera PM595 (FLIR, USA). The lid of the respirometry chamber was removed and images were recorded at a height of 1 m above the chamber. Thermal images were analysed using Thermacam Reporter 7.0 (FLIR, USA) using an emissivity for fur of 0.98 [29]. Surface temperatures were averaged from images taken at start and end of the respirometry periods. The mean surface temperature of pups was determined by fitting a polygon around the individual animal in the case of isolated pups and around the entire huddle for G6. We chose to measure three sites of surface temperatures: back, ear and brown adipose tissue (BAT). As a response to cold, vasoconstriction was investigated by measuring ear temperature (T_ear_), the pinna being the principal site of heat dissipation for rabbits [30]. Heat production through non-shivering thermogenesis was estimated measuring BAT surface temperature (T_BAT_). The back temperature (T_back_) therefore represented a reference surface temperature, from a neutral area with respect to heat flow. The gradient (T_BAT_ - T_back_) allowed the comparison of BAT activation independently of ambient temperatures (23 or 14°C). For G1 pups, surface temperatures (back, ear and BAT) were calculated as the mean (± SD) of two individuals for each age group. For G6 pups, the mean (± SD) surface temperatures (back, ear and BAT) were determined from three individuals within the group as positions of pups prevented a clear view of all pups within the huddle. The back region was defined using a circle (diameter = 20 pixels) positioned on the dorsal surface in the centre of the hips. Ear temperatures were determined by fitting a polygon around the outer edge of both left and right ears. As it was not possible to precisely define the surface overlying BAT, a circle (diameter = 20 pixels) was positioned on the image between scapulae (following [31], [32]).

### Statistical analyses

Prior to analyses, we verified on raw data the absence of any difference between litters and categories of pups.

Cold challenges in the chambers were tested independently for the four categories of pups, between the four ambient temperatures, using ANOVAs and Kruskal-Wallis ANOVAs when distribution was not normal. In order to compare local heating between G6 and G2 pups, insulated and non-insulated pups were pooled and Mann-Whitney tests were used for each room temperature. Body mass of pups were compared with paired t-tests between the two sessions: G6 pups at day 4, and G2 pups at day 5; G6 pups at day 15, and G2 pups at day 16. To assess the effects of cold challenge, huddling (G2 vs. G6), and age (non-insulated vs. insulated pups) on either energy expenditure or body temperature, and to take into account repeated measurement nature of the data, multivariate linear mixed-effects regression models were implemented using the SAS PROC MIXED procedure (SAS Institute, Cary, NC). Independent variables included external temperature, group (G2 vs. G6), and age (insulated vs. non-insulated). Body mass was also included as a potential confounder. These models were fitted to the data with the intercept as the random effect.

Surface temperature of the different tissues (ear, back and BAT for isolated pups; n = 4, and ear, back and BAT for pups in groups of 6; n = 6) were compared with one-way ANOVAs on repeated measures for each category (post-hoc Tukey tests).

Results are expressed as means ± SD. Statistical tests are considered significant at p<0.05.

## Results

### How does the huddle determine the thermal environment and physiological responses of pups?

#### Comparison of cold challenges experienced by all categories of pups

Room temperature during the cold challenge was decreased successively from 22.8±0.8 to 18.2±0.8, 15.0±0.8, and 10.6±1.3°C (**Table 1**). Decreasing ambient temperatures in the experimental room did not differ between groups (non-insulated and insulated, G6 and G2 pups, p>0.08 for 23, 18, 15 and 11°C). Cold challenges inside the boxes were similar for the four categories of pups (G6 and G2, non-insulated and insulated pups compared at 23, 18, 15 and 11°C: H = 8.938, p = 0.03, no post hoc differences for 23°C; p>0.12 for other temperatures; **Table 1**). However, by pooling cold challenges of insulated and non-insulated pups grouped by six or two, local heating for G6 pups was significant at 18°C (+1.4°C compared with G2 pups, U = 154, p = 0.05), and close to significance at 15°C (+1.8°C, U = 149, p = 0.085) and at 11°C (+2.4°C, U = 150, p = 0.076; **Table 1**). Local heating was not significant at 23°C (U = 139.5, p = 0.202). Hence, due to technical constraints imposed by respirometry measurements, G6 pups (insulated and non-insulated) were exposed locally to slightly less of a cold challenge in comparison to G2 pups.

**Table 1:** Mean (± SD) ambient temperatures experienced by different categories of pup groups in the chambers (G6 or G2 pups, non-insulated and insulated) as a function of the cold challenge in the experimental room.

	G6 non-insulated	G6 insulated	G6	G2 non-insulated	G2 insulated	G2
Room temperature (°C)						
22.8±0.8	24.7±0.9	23.4±1.9	**24.0±1.4**	23.8±0.3	22.9±0.9	**23.4±0.6**
18.2±0.8	20.8±1.2	20.2±2.4	**20.4±1.9**	19.2±1.4	18.8±1.1	**19.0±1.3**
15.0±0.8	18.4±2.4	17.1±3.6	**17.6±3.0**	16.0±2.0	15.5±1.9	**15.8±1.9**
10.6±1.3	14.4±2.8	13.1±4.3	**13.7±3.5**	11.6±2.5	10.9±2.4	**11.3±2.4**

Between the two sessions (at day 4 and day 5), pups gained on average 13.4±5.3 g, corresponding to a 14% increase in body mass (body mass of G6 pups at day 4: 98.9±15.9 g, of G2 pups at day 5: 112.3±18.9 g, t = −12.3, p<0.001). In the same way, between day 15 and day 16, pups gained 15.2±7.0 g, i.e. 6% of their total body mass (body mass of G6 pups at day 15: 267.6±41.7 g, of G2 pups at day 16: 282.8±43.5 g, t = −10.6, p<0.001). Hence, G2 pups, one day older than when placed in groups of six, possessed a higher body mass, i.e. a lower surface area to volume ratio than G6 pups. This may have minimised any physiological variations and effects linked to huddling.

#### Physiological responses to cold for non-huddling (G2 pups) *vs.* huddling (G6 pups) and non-insulated *vs.* insulated pups (Figures 1A & 1B)

**Figure 1:**
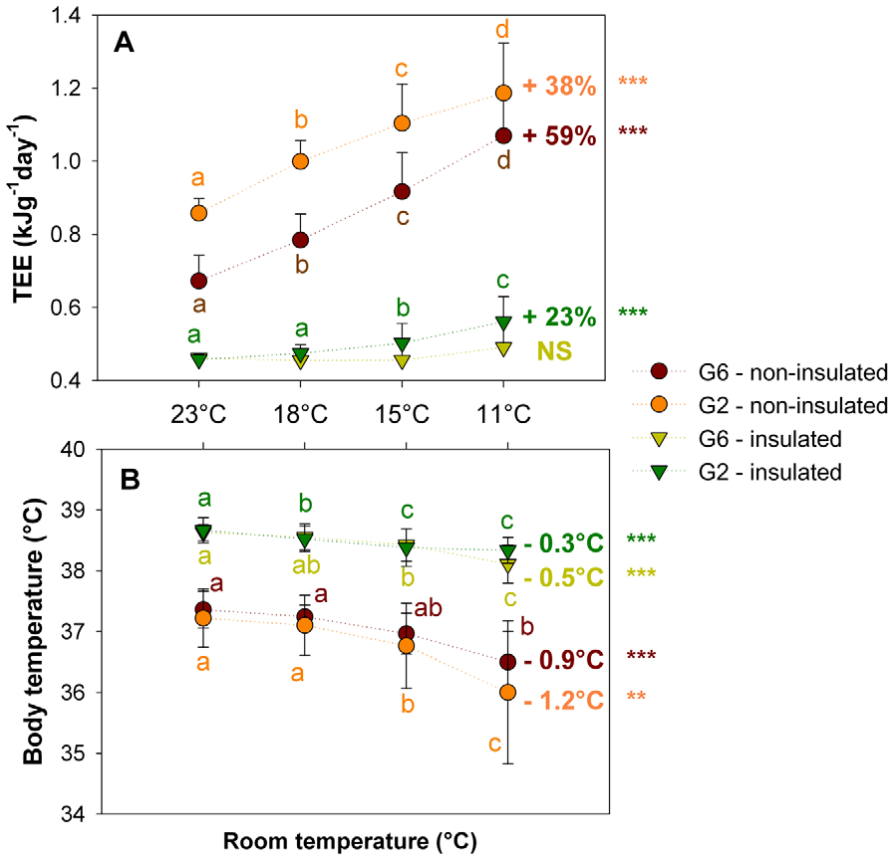
Relationship between total energy expenditure (TEE) and body temperature of insulated and non-insulated pups, huddling in groups of 6 (G6) or placed in groups of 2 (G2), during the cold challenge. Different letters indicate significant differences. ***: significant differences with p<0.0001 between 23°C and 11°C; **: significant differences with p<0.002 between 23°C and 11°C. NS: not significant.

For all ambient temperatures, non-insulated pups showed a total energy expenditure (TEE) more than twofold higher than insulated pups (0.99±0.17 *vs.* 0.49±0.06 kJ.g^−1^.day^−1^; p<0.001 after adjustment for cold, group and body mass). In addition, TEE of G6 pups was 14% lower than G2 pups (0.66±0.24 *vs.* 0.77±0.29 kJ.g^−1^.day^−1^; p = 0.02 after adjustment for cold, age and body mass).

TEE of non-insulated pups (**Figures 1A & 1B**), both in G6 and G2, started to increase significantly at 18°C, and further at 15 and 11°C (p<0.004 for all temperatures after adjustment for body mass). While body temperature of G2 pups started to decrease significantly at 15°C (p = 0.04 after adjustment for body mass between 18°C and 15°C), body temperature of G6 pups started to decrease at the lower temperature of 11°C (p<0.001 after adjustment for body mass between 18°C and 23°C and 11°C).

Between the two extreme ambient temperatures of 23°C and 11°C, TEE of non-insulated G6 pups significantly increased by 59%, while their body temperature decreased by 0.9°C (p<0.0001 and p = 0.0015 respectively, after adjustment for body mass). In the same way, TEE of non-insulated G2 pups increased significantly by 38% in response to cold, while their body temperature decreased by 1.2°C (both p<0.0001 after adjustment for body mass).

Considering insulated pups (**Figures 1A & 1B**), TEE of G6 pups did not increase in response to cold (p>0.13 for all temperature after adjustment for body mass). TEE of G2 insulated pups increased when exposed to lower temperatures of 15 and 11°C (p<0.05 after adjustment for body mass). In contrast, body temperature of both G6 and G2 pups started to decrease significantly at 15°C (p = 0.0045 for G6 and p<0.0001 for G2 pups after adjustment for body mass).

Between the two extreme ambient temperatures of 23°C and 11°C, TEE of G6 insulated pups increased non significantly by 6%, while their body temperature decreased by 0.5°C (p = 0.226 and p<0.0001 respectively, after adjustment for body mass). By contrast, TEE of G2 insulated pups increased by 23% in response to cold, while their body temperature decreased by 0.3°C (both p<0.0001 after adjustment for body mass).

### Brown adipose tissue thermogenesis of non-insulated and insulated pups exposed to cold while huddling (G6) or isolated (G1): how do individual pups contribute to group thermoregulatory processes in the huddle?

**Figure 2:**
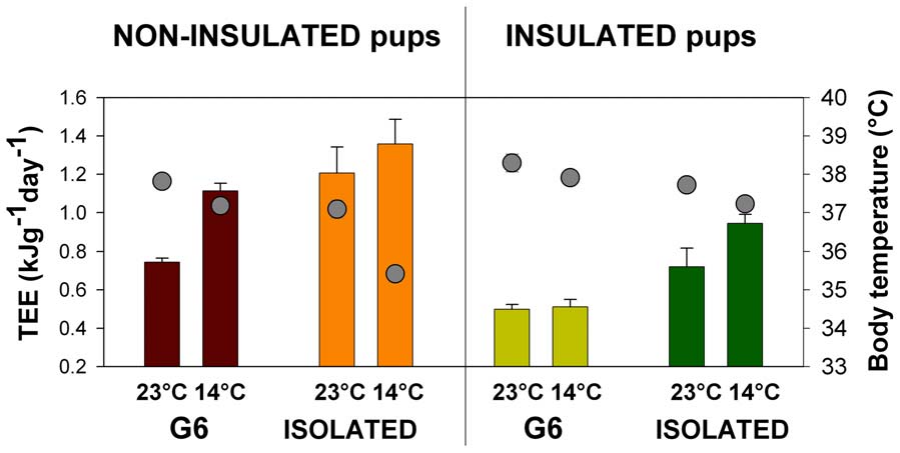
Total energy expenditure (TEE, kJg^−1^day^−1^; bars) and body temperature (°C; circles) for each pup category (G6 huddling and G1 isolated pups, insulated and non-insulated, exposed at 23°C and 14°C).

Body temperature and energy expenditure responses of G6 and G1 pups, at 23°C (23.0±0.1°C) and 14°C (14.0±0.7°C) for non-insulated (at 3 and 4 days old) and insulated pups (at 10 and 11 days old), were consistent with Experiment 1 (**Figure 2**). The mean (± SD) mass of G6 pups aged 3 days was 98.7±5.9 g, while G1 pups aged 4 days was 108.9±4.7 g, representing a 10% increase in body mass. For insulated pups, G1 pups aged 10 days averaged 215.5±57.2 g, while the mass of G6 pups aged 11 days was 229.4±34.3 g, thus representing a 6.5% increase in body mass.

Ambient temperatures were monitored in each experimental chamber. At warm ambient temperature, local heating in the chambers containing G6 pups compared with isolated pups was 0.8°C for non-insulated pups (24.4±0.1°C for 3-day G6 pups *vs.* 23.6±0.3°C for 4-day G1 pups) and 2.1°C for insulated pups (25.6±0.6°C for 11-day G6 pups *vs.* 23.5±0.1°C for 10-day G1 pups). In the same way, at cold ambient temperature, local heating was 3.7°C for G6 non-insulated pups (18.0±0.2°C for 3-day G6 pups *vs.* 14.3±0.4°C for 4-day G1 pups) and 2.6°C for G6 insulated pups (18.2±0.3°C for 11-day G6 pups *vs.* 15.6±0.3°C for 10-day G1 pups). Cold challenges were therefore 6.4°C for 3-day G6 pups, 9.3°C for 4-day G1 pups, 7.4°C for 11-day G6 pups, and 7.9°C for 10-day G1 pups.

#### BAT activation and ear vasoconstriction: comparison of BAT, ear, and back surface temperatures

Considering non-insulated pups, at warm ambient temperatures, the mean surface temperature of the huddle (i.e. group of 6 pups) was 1.9°C higher than the mean temperature of isolated pups (36.9±0.9°C *vs.* 35.0±0.5, respectively). At cold ambient temperatures, the mean surface temperature of the huddle was 4.7°C higher than the mean temperature of isolated pups (35.6±0.4 *vs.* 30.9±0.9°C).

**Figure 3:**
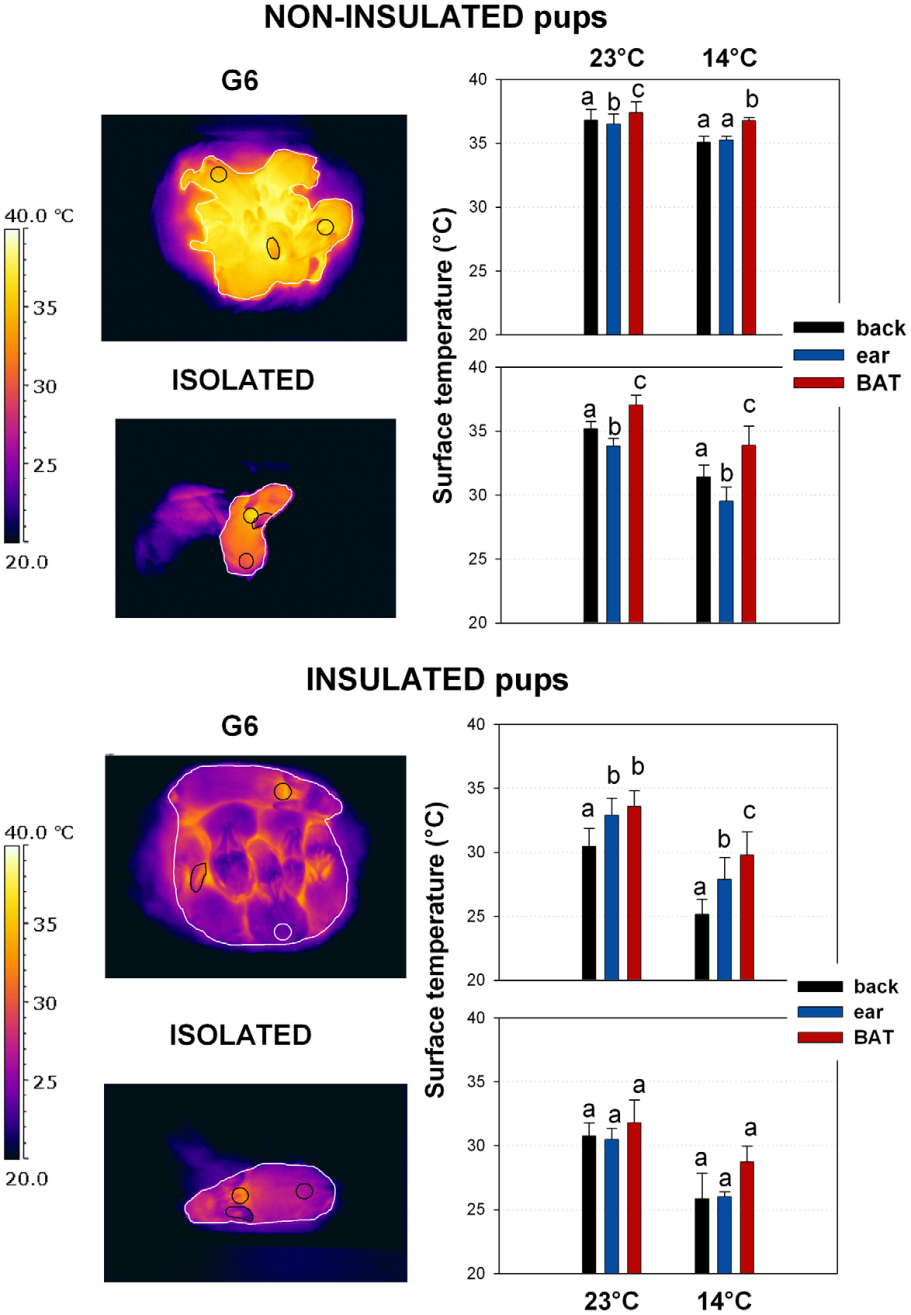
Surface temperature of back, ear and brown adipose tissue (BAT) of isolated (G1) and huddling (G6) pups, non-insulated and insulated, exposed at 23°C and 14°C. The results of post-hoc Tukey tests are shown by the letters a to c: same letter showed no significant difference. Left panel: Thermal images are shown for isolated (G1) and huddling (G6) non-insulated and insulated pups, exposed to an ambient temperature of 14°C. Images show the location of circles used to compare surface temperature (total area, back, ear, brown adipose tissue (BAT)).

BAT surface temperature was significantly higher than back and ear surface temperatures for each category of non-insulated pup (p<0.001 in all cases, post-hoc p<0.05; **Figure 3**). Back surface temperatures were also significantly higher than ear surface temperatures (post-hoc p<0.05), except for G6 non-insulated pups at 14°C (post-hoc test non significant; **Figure 3**).

Considering insulated pups, at 23°C, the mean surface temperature of the huddle was 1.2°C higher than the mean temperature of isolated pups (32.0±0.8°C *vs.* 30.8±0.8, respectively). At 14°C, the mean surface temperature of the huddle was 0.9°C higher than the mean temperature of isolated pups (26.7±0.4 *vs.* 25.8±1.0°C).

The mean BAT surface temperature was higher than the mean back surface temperature of G6 insulated pups (p<0.001 in both cases; **Figure 3**). However, BAT surface temperature of G1 insulated pups was not significantly different from back and ear temperatures (p>0.05 in both cases; **Figure 3**).

#### BAT activation, body temperature and energy expenditure

**Figure 4:**
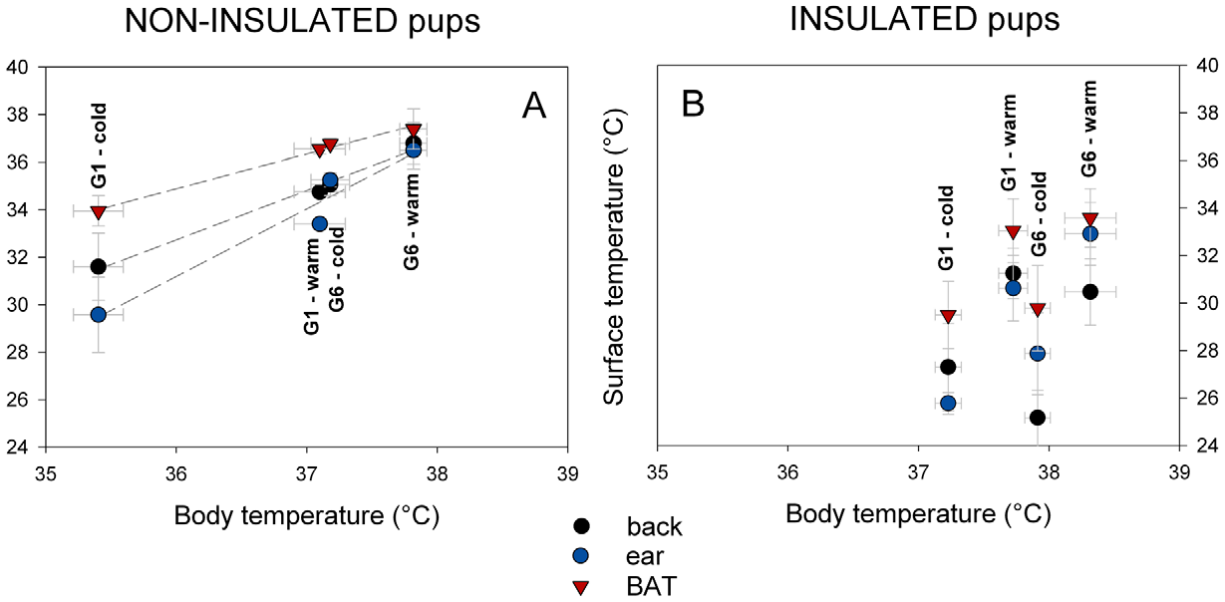
Surface temperature of back, ear and brown adipose tissue (BAT) of non-insulated (**Figure 5A**) and insulated (**Figure 5B**) pups (isolated, G1 or huddling, G6) as a function of their body temperature. Regression lines are statistically significant for non-insulated pups only. Non-insulated pups: T_BAT_ = −17.79+1.46*T_b_, r^2^ = 0.992, p = 0.004; T_ear_ = −71.46+2.85*T_b_, r^2^ = 0.950, p = 0.025; T_back_ = −42.14+2.08*T_b_, r^2^ = 0.988, p = 0.006. Insulated pups: T_BAT_, r^2^ = 0.436, p = 0.340; T_ear_, r^2^ = 0.732, p = 0.144; T_back_, r^2^ = 0.086, p = 0.707.

BAT, ear, and back surface temperatures were correlated with body temperature for non-insulated pups (R^2^≥0.95, p≤0.025 in all cases **Figure 4A**). By contrast, BAT, ear, and back surface temperatures of insulated pups were not correlated with body temperature (R^2^≤0.73, p>0.14 in all cases **Figure 4B**).

**Figure 5:**
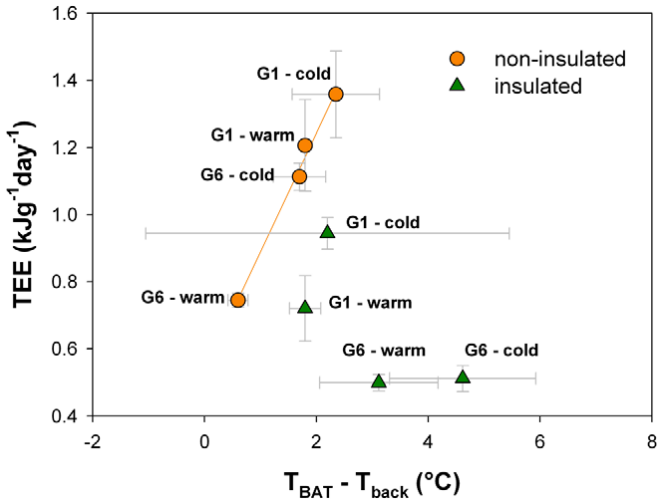
Total energy expenditure (TEE) as a function of surface temperature difference between brown adipose tissue (T_BAT_) and back (T_back_). G1 stands for isolated pups and G6 for huddling pups, non-insulated (circle) or insulated (triangle). Regression line is statistically significant for non-insulated pups only: TEE = 0.534+0.354*(T_BAT_ - T_back_), r^2^ = 0.991, F_1,3_ = 223.9, p = 0.004.

In order to determine whether BAT surface temperature, T_BAT_, could be used as an index of energy expenditure, the relationship between total energy expenditure (TEE) and T_BAT_ - T_back_, at warm and cold temperatures was examined (**Figure 5**). For non-insulated pups, TEE was highly correlated with T_BAT_ - T_back_ (TEE = 0.534+0.354.(T_BAT_ - T_back_), r^2^ = 0.991, F_1,3_ = 223.9, p = 0.004; **Figure 5**). However, for insulated pups, TEE was not correlated with T_BAT_ - T_back_ (r^2^ = 0.500, p = 0.293; **Figure 5**).

## Discussion

Cold challenges (from 23 to 11°C) were designed to be significant for developing rabbit pups [4]–[6], [18], [19]. Due to experimental constraints, rabbit pups were cold challenged at 4 and 15 days old in G6 (huddling condition) and a day later in G2 (non-huddling condition). In a second experiment, G6 pups were cold challenged at 3 and 11 days old and isolated (G1) pups were challenged at 4 and 10 days old. This difference in age may have weakened the comparison between huddling and non-huddling pups since they did not possess strictly similar heat loss constraints linked to their different body mass, and hence their different surface to volume ratio [33], [34]. As non-insulated pups when huddling possessed less favourable heat loss constraints, this procedure reinforces our findings with respect to energy savings associated with huddling. The comparison between huddling and non-huddling pups however remained valid for non-insulated pups (up to 5 days old, when they have no or little fur and a high surface to volume ratio), and insulated pups (from 5 days old, when their body mass has increased and they have grown fur; [6]). According to Alberts' analysis [10], the experimental design may have actually strengthened the comparison between huddling and non-huddling animals by avoiding physiological differences between individuals.

### Huddling promotes local heating and reduces the cold challenge

The chambers were closed for respirometry measurements, and therefore local heating in the chambers containing G6 huddling pups was important in comparison to isolated or G2 pups. In warm conditions (23°C), local heating was 0.8°C higher for huddling non-insulated pups (aged 3–4 days), and 2.1°C higher for huddling insulated pups (aged 10–11 days) compared with isolated littermates. Local heating was even more pronounced in a cold environment (14°C): surrounding temperatures of huddling pups were 3.7 and 2.6°C higher compared with isolated pups (non-insulated and insulated pups, respectively). Warming of the local microclimate is part of the thermal benefits of huddling [8]. For example, a 5°C increase in ambient temperature within the nest of huddling short-tailed field voles (*Microtus agrestis*) accounts for 55% of the energetic benefits of huddling [35]. Bautista *et al*. [5] showed that the ambient temperature in the immediate vicinity of groups of four to six huddling rabbit pups was 26°C, significantly higher than for groups of two (24°C) or for isolated pups (23°C) when pups were exposed to a similar ambient temperature. Hence single pups or pairs effectively experienced colder conditions compared with pups huddling in a group of six. Local heating moreover directly impacted on the mean surface body temperature of the pups. The surface temperature of huddling non-insulated pups was 1.9 and 4.7°C higher than isolated pups in warm and cold conditions, respectively. When insulated, the surface temperature of huddling pups was 1.2 and 0.9°C higher than isolated littermates when pups were exposed to warm and cold conditions, respectively. Our results therefore show that local heating is crucial in reducing the extent of the cold challenge especially in the first days of life, when pups experience thermal stress. This may be frequent for rabbit pups as unlike many other mammals, female rabbits leave their pups soon after birth, and only nurse them for 3 to 5 min once a day [36]–[38]. The thermal environment of pups depends both on the insulative properties of the nest and of the heat produced by each pup within the litter. The huddle therefore provides for each individual a “public” warmth that is beneficial to survival, especially in the cold and when pups are non-insulated.

### Huddling acts as a buffer for newborns to delay thermogenic responses to the cold

When exposed to cold, rabbit pups increase their energy expenditure by a rise in metabolic heat production but when overwhelmed by heat loss body temperature decreases [4], [6]. In this study huddling pups showed a delayed thermogenic response compared with their non-huddling littermates when faced with a progressive cold challenge (from 23 down to 11°C). When non-insulated, the increase in energy expenditure started when both huddling and non-huddling pups were exposed at 18°C. At 11°C, energy expenditure increased by 59% for huddling pups and 38% for non-huddling pups. However, the decrease in body temperature was delayed and less pronounced for huddling pups (significant drops of 0.5°C at 15°C and 1.2°C at 11°C for non-huddling pups compared with a 0.9°C drop at 11°C for huddling pups). When insulated, energy expenditure increased for non-huddling pups by 23% at 11°C but this did not occur when pups were huddling. Similarly, the body temperature of insulated non-huddling pups decreased at 18°C, while this only occurred for huddling pups at 15°C. Insulated huddling pups, exposed to the same ambient conditions did not appear to face any cold challenge.

In endotherms, physiological responses to cold involve vasoconstriction to reduce skin surface temperature and thermoregulatory thermogenesis to maintain homeostasis [33]. In newborns, thermogenesis primarily occurs through non-shivering thermogenesis (NST) with BAT activation [4]. Interscapular and cervical BAT is functional at birth in rabbits [18], [19], [39]. As temperature influences cellular metabolism [33] and hence growth, maintenance of a high body temperature is an especially important factor shaping development. Any delay in thermal responses due to huddling reduces energy requirements and promotes pup growth. These results also highlight that a higher and less variable body temperature, favourable to growth, is more easily maintained by huddling individuals (in rabbits [5], [6]; in rats [10]; for review [8]). Indeed, Bautista *et al.* [11] found a positive relationship between huddling behaviour of individuals and their body temperature within the litter.

Within their litter, altricial mammal pups cooperate for warmth, but compete for energy, obtained from maternal milk and allocated to growth and thermogenesis. Pup growth in rabbits is hence primarily dependent on maternal factors and litter size [7], [40]. Food provisioning for an individual rabbit pup in a litter is indeed limited by maternal milk production [7], [40], [41] and its competitiveness to access the mother's teats during the brief daily suckling period [38], [42], [43], [44]. Moreover, survival is influenced by the pups' body mass [7] and the amount of energy allocated to growth is dependent on the amount of energy lost for thermoregulation, linked to heat loss [6]. In wild rabbits the thermal environment during development is an important determinant of optimal litter size [7], through the trade-off between a limited energy supply (milk) and the cooperative warmth gained by pups huddling in the litter. The results of our study suggest that altricial rabbit pups are proximately constrained by the cost of thermoregulation but that this is alleviated by the benefits of huddling. Huddling indeed delays the onset of thermogenesis by providing “public warmth” that influences the cost of thermoregulation for each individual.

### Huddling reduces individual BAT heat production

**Figure 6:**
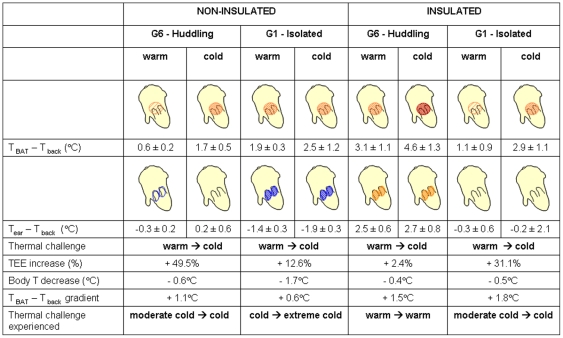
Gradients between brown adipose tissue (BAT) and back surface temperatures (°C), and ear and back surface temperatures (°C) for each pup category (G6 - huddling, G1 - isolated, non-insulated and insulated pups). The percentage increase in total Energy Expenditure (TEE) is based on Figure 2. Orange and red colours represent BAT and ear surface temperatures higher than back surface temperature (i.e. heat production or vasodilatation). Blue colours represent ear vasoconstriction. Denser shading represents higher surface temperature gradients.

When non-insulated, both huddling (G6) and isolated pups produced extra heat when exposed to cold: their energy expenditure increased, while their body temperature decreased (**Figure 2**). Isolated pups were however faced with a higher cold challenge than huddling pups as their capacity to thermoregulate was exceeded. For non insulated pups the increase in TEE between warm and cold conditions was 49.5% for huddling pups, and 12.6% for isolated pups (**Figure 6**). Even in warm conditions these isolated pups increased metabolic heat production for effective thermoregulation. However, when exposed to cold isolated pups were only able to increase thermogenesis by a relatively small amount, resulting in a drop in body temperature of 1.7°C. Furthermore, thermal images showed that T_BAT_ was higher than T_back_ in all cases, indicating that heat production was largely due to BAT activation. Pups also reduced their heat loss by vasoconstriction, as seen by a drop in ear temperature (**Figure 6**). As BAT, ear, and back surface temperatures were correlated with body temperature, both for huddling and isolated pups in warm and cold conditions, the heat produced by non-shivering thermogenesis through BAT is therefore required for the maintenance of a stable and high body temperature. Previously, infrared thermography provided a useful tool to reveal BAT activation [4], [21], [31], [32], [45], [46]. As revealed by thermal images, it is known that the thermogenic capacity of BAT is determined by behavioural modulation of huddling in infant rats [4], [20], [22], [47]. In addition, Oya *et al.* [46] reported that non-shivering thermogenesis (NST) is enhanced through BAT activity after birth in newborn humans. Other authors [31], [45] showed that ultrasonic vocalizations and thermogenesis are linked when rat pups are isolated and exposed to various ambient conditions. Sokoloff and Blumberg [21] moreover investigated competition and cooperation among litters of rat pups by inhibiting BAT thermogenesis for 0, 2, or 4 rats in each huddle. Inhibition of BAT thermogenesis, revealed by IRT, indeed compromised the ability to maintain huddle temperature during a cold exposure. Jackson *et al.* [32] however attempted to use IRT to correlate changes in BAT temperature with energy expenditure. More precisely, these authors attempted to quantify NST in BAT of short-tailed field voles (cold-acclimatized or not), following a noradrenaline injection, a BAT activator. They could not find any significant correlation between changes in surface temperature and the metabolic peak associated with the noradrenaline injection. However in our study, TEE was highly correlated with the gradient T_BAT_ - T_back_, indicating that the extent of BAT non-shivering thermogenesis directly influenced the energy expenditure of the pup.

The ambient temperatures selected in our procedure were challenging both for huddling and non-huddling non-insulated pups (also see [18], [19], [4]–[6]). The cold treatment was however too challenging for isolated pups as heat loss overwhelmed heat production. To compare the thermogenic responses of insulated pups (older than 5 days) with those of non-insulated pups (from birth up to 5 days old), we chose to select similar ambient temperatures for older pups. However, as pups develop they increase their body mass (decrease their surface to volume ratio) and grow fur (increase their insulation capacities), their thermoregulatory constraints and their thermoneutral zone change [4]. Hence, the tested cold challenge was not sufficient for huddling insulated pups: they were paradoxically placed in a situation of warm challenge instead of cold challenge, notably due to the high efficiency of huddling and local heating. Indeed, their ear temperature exceeded their back temperature (**Figure 6**). Since only isolated insulated pups were cold challenged, while huddling insulated pups were warm challenged, none of the correlations for non-insulated pups were found significant for the older categories. Body temperature showed no correlation with surface temperatures, and in the same way TEE was not correlated with the gradient T_BAT_ - T_back_. However, when comparing isolated insulated pups at warm and cold temperatures, they seem to present thermogenic responses similar to non-insulated pups (**Figure 5** **&** **6**), even though they maintained a higher body temperature and possessed lower surface temperatures, presumably due to greater insulation.

As energy supply is limited for pups, extra energy required for thermally challenged pups will reduce energy available for growth and possibly influence their short or long-term survival and their adult performances [40], [48]–[50]. In the cold (14°C), non-insulated huddling pups had body and surface temperatures that were equivalent to their isolated littermates exposed to a warm ambient temperature (23°C, **Figure 4A**). Moreover, in a warm environment, non-insulated huddling pups maintained a TEE equivalent to older insulated pups that were isolated (**Figure 5**). The surface temperature gradients (T_BAT_ - T_back_ and T_ear_ - T_back_) of non-insulated huddling pups were equivalent to insulated isolated individuals (23°C, **Figure 6**). Hence, even when pups are younger than 5 days old, and non-insulated, huddling provides them “public insulation”. Huddling non-insulated pups then reduce their energy requirements, switching from a “non-insulated state” to a pseudo “insulated state”, thanks to the reduction in their cold-exposed body surface and the local heating provided by huddling. Huddling therefore drastically modifies thermal constraints of pups, reducing the cold challenge and effectively increasing their developmental stage of thermoregulation.

Huddling reduces BAT thermogenesis, allowing pups to lower the amount of “private heat” given to the “public good”, essential for the maximum allocation of energy for growth. When ambient temperatures were challenging, the pattern of thermogenic responses was equivalent when pups were exposed to warm or cold temperatures in our study, but differed considering the extent of the response (**Figure 5**). Moreover, for cold challenging conditions, huddling pups responded similarly to isolated pups (significant regression line, **Figure 5**). Huddling therefore did not seem to modify the individuals' thermogenic response, but the extent of their response, and hence the extent of the energy allocated to maintenance of their body temperature. Considering these results, we may assume that all pups invested to the same extent in the public good, and that presumably no selective forces drive some selfish less-related pups within a litter to invest less “private heat” into the “public warmth”. In rat pups, Sokoloff and Blumberg [21] showed that the inhibition of BAT thermogenesis compromised the ability of pups to maintain huddle temperature, but this did not result in enhanced huddling. They concluded that the heat provided by BAT appeared to shape behavioural interactions in the huddle during development, since effective huddling during cold exposure requires the thermal resources provided by BAT activation. In addition, the ability of individuals to obtain access to warmth within a huddle is not related to birth weight, survival, milk intake, or metabolic efficiency [11]. It appears from these studies that neonates share out thermally advantageous positions rather than compete within the huddle, as they continually move through it. However, a recent study shows that positions of pups within the huddle could possibly be linked to different personalities [51], and hence would depend on individual physiological and behavioural characteristics. Given that it may be possible to explore the selection of maternal and paternal genes controlling BAT thermogenesis [12], further studies examining the behaviour and physiological performances of different individuals within a litter may reveal new insights into the extent to which huddling is altruistic, and to investigate proximal factors that drive the development of individual personality [52].

### Conclusions

Proximal thermoregulatory constraints that ultimately govern development and survival of rabbit pups were examined. Huddling, through “public warmth”, drastically modifies thermal constraints of pups, reducing cold challenges and effectively increasing their developmental stage of thermoregulation. Since energy is limited for an organism, variations in its thermoregulatory requirements impact on the amount of energy allocated to growth. By providing public warmth when pups are the most vulnerable, huddling buffers cold challenges and ensures a constant allocation of energy to growth by delaying non-shivering thermogenesis. This study may stimulate further research focusing on the energetic implications of cold challenges in other altricial infants, and more particularly by investigating individual thermogenic constraints within a litter on the early development of personality differences between siblings.
